# Supplementary material for: Determination of Biological and Molecular Attributes Related to Polystyrene Microplastic-Induced Reproductive Toxicity and Its Reversibility in Male Mice
Source: Int J Environ Res Public Health. 2022 Oct 28;19(21):14093. doi: 10.3390/ijerph192114093 (PMC9656262; doi:10.3390/ijerph192114093)
Supplement: Supplementary file 1 [file ijerph-19-14093-s001.zip › ijerph-1936701-supplementary.pdf]

Kurt particle size analysis of polystyrene microplastics

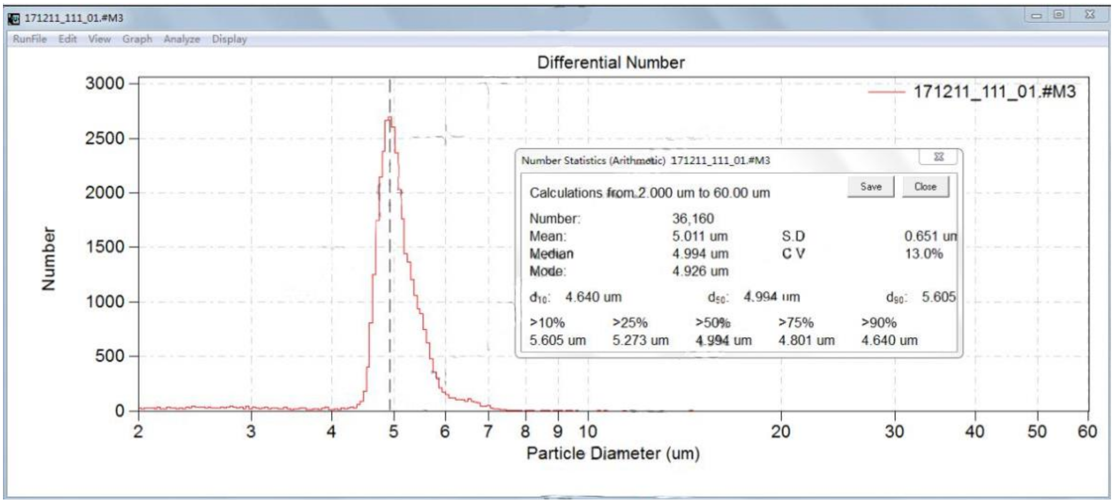

Figure S1. Kurt particle size analysis of polystyrene microplastics. The figure shows that the particle size is concentrated at 5 μm, mean±SD（5.011±0.651）μm.

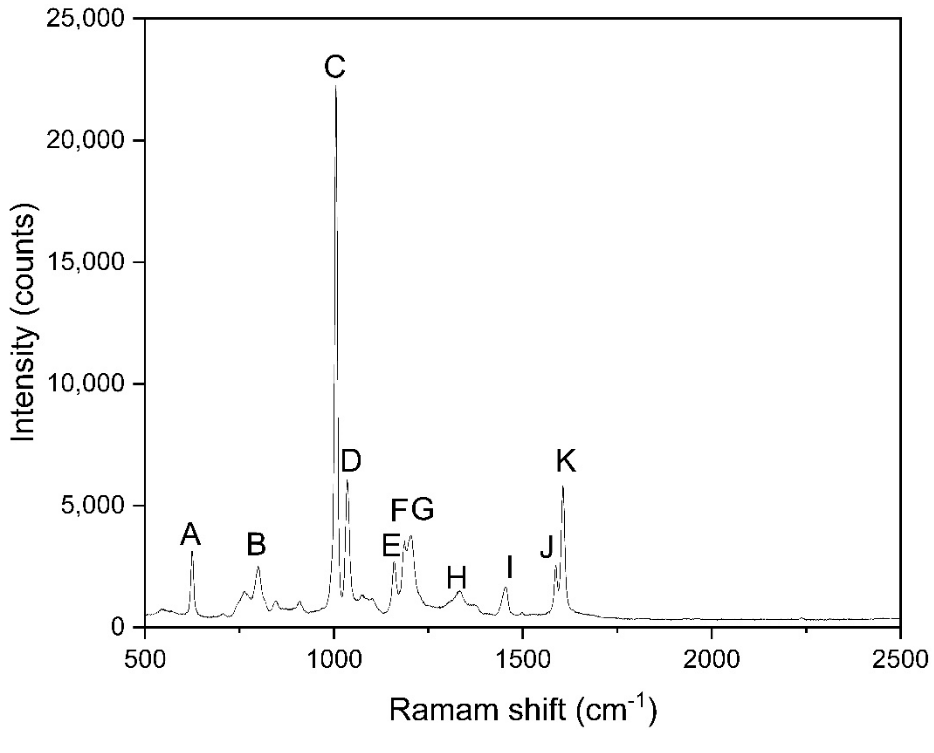

Figure S2. The spectral characteristic peaks are concentrated in the region of 500~2000cm^{-1}, and show strong intensity in the characteristic bands of A, B, C, D, E, F, G, H, I, G and K. A: 626 cm^{-1}, B: 804cm^{-1}, C: 1006cm^{-1}, D: 1037cm^{-1}, E: 1158cm^{-1}, F: 1186cm^{-1}, G: 1202cm^{-1}, H: 1328cm^{-1}, I: 1452cm^{-1}, J: 1586cm^{-1}, K: 1606cm^{-1}.

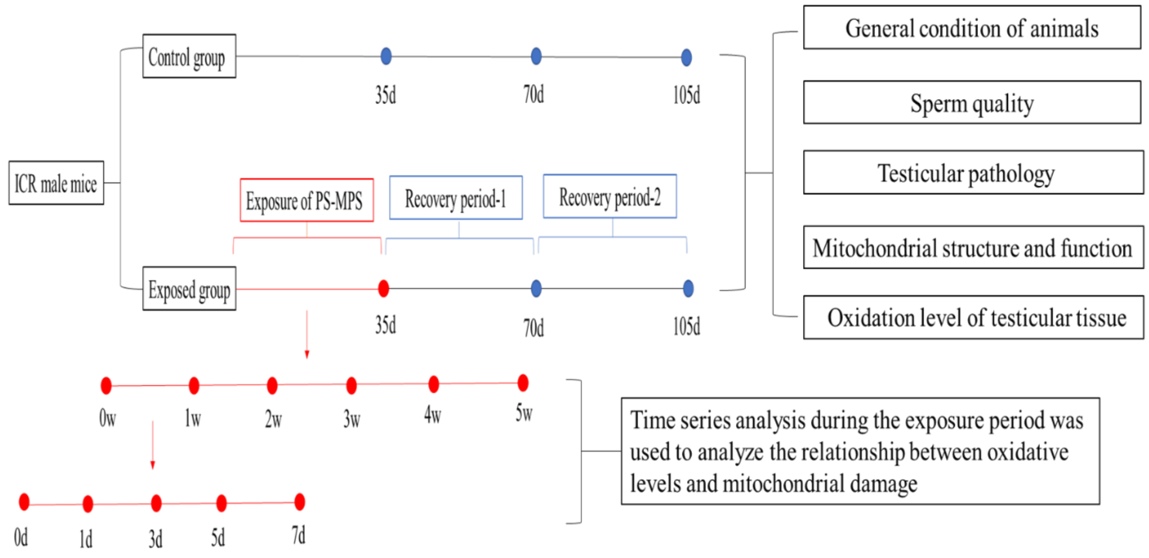

Figure S3. Presentation of the study design.

Table S1. Primer sequences of PCR

Primers	Sequence
long fragment	F: 5'-GCCAGCCTGACCCATAGCCATAATAT-3'
	R: 5'-GAGAGATTTTATGGGTGTAATGCGG-3'
short fragment	F: 5'-CCCAGCTACTACCATCATTCAAGT-3'
	R: 5'-GATGGTTTGGGAGATTGGTTGATGT-3'

Table S2. Primer sequences of fluorescence quantitative PCR

Primers	Sequence
OPA1	F: 5'-TGGAAAATGGTTCGAGAGTCAG-3'
	R: 5'-CATTCCGTCTCTAGGTTAAAGCG-3'
Drp1	F: 5'-ACCGGGAATGACCAAAGTACC-3'
	R:5'-TGGGATTACTGATGAACCGAAGA-3'
PINK1	F: 5'-GGCTTCCGTCTGGAGGATTAT-3'
	R: 5'-AACCTGCCGAGATATTCCACA-3'
Parkin	F: 5'-GGTCCTACAGACAGGGCAATA-3'
	R: 5'-CTGGCCTTTCCTCACACCAC-3'
β-actin	F: 5'-GTGACGTTGACATCCGTAAAGA-3'
	R: 5'-GCCGGACTCATCGTACTCC-3'
COX1	F: 5'-TCGCCATCATATTCGTAGGAG-3 '
	R: 5'-GTAGCGTCGTGGTATTCCTGA-3'
GAPDH	F: 5'-GAGGGGCCATCCACAGTCTTC-3'
	R: 5'-CATCACCATCTTCCAGGAGCG-3 '

Table S3. Organ weight and coefficient after the exposure period

	Control group		Exposed group	
Organ	Weight(g)	Coefficient (%)	Weight(g)	Coefficient (%)
Heart	0.24±0.02	0.55±0.05	0.27±0.05	0.59±0.1
Liver	2.34±0.24	5.41±0.54	2.32±0.19	5.19±0.46
Spleen	0.18±0.09	0.31±0.05	0.15±0.01	0.33±0.03
Lung	0.29±0.05	0.61±0.12	0.28±0.05	0.63±0.11
Kidney	0.7±0.05	1.61±0.15	0.75±0.06	1.69±0.11
Testis	0.3±0.04	0.61±0.14	0.29±0.02	0.66±0.04

Table S4. Organ weight and coefficient after the first recovery period

	Control group		Exposed group	
Organ	Weight(g)	Coefficient(%)	Weight(g)	Coefficient(%)
Heart	0.28±0.05	0.6±0.13	0.28±0.04	0.59±0.1
Liver	2.33±0.25	4.98±0.33	2.28±0.61	4.79±1.17
Spleen	0.16±0.03	0.33±0.06	0.17±0.05	0.35±0.08
Lung	0.31±0.04	0.67±0.11	0.31±0.05	0.66±0.12
Kidney	0.76±0.1	1.61±0.15	0.76±0.07	1.62±0.19
Testis	0.28±0.05	0.6±0.09	0.29±0.05	0.62±0.13

Table S5. Organ weight and coefficient after the second recovery period

	Control group		Exposed group	
Organ	Weight(g)	Coefficient(%)	Weight(g)	Coefficient(%)
Heart	0.31±0.07	0.65±0.15	0.28±0.05	0.58±0.09
Liver	2.72±0.38	5.76±0.54	2.55±0.28	5.38±0.25
Spleen	0.17±0.02	0.37±0.04	0.15±0.06	0.33±0.16
Lung	0.36±0.08	0.75±0.15	0.3±0.04	0.64±0.1
Kidney	0.85±0.14	1.78±0.25	0.86±0.18	1.82±0.29
Testis	0.27±0.05	0.57±0.11	0.3±0.05	0.64±0.09

Table S6. Abbreviations and acronyms

English abbreviations	Complete spelling
ATP	Adenosine triphosphate
DHE	Dihydroethidium
HE	*He*matoxylin-eosin
HO-1	Heme Oxygenase-1
IL-1β	Interleukin-1β
IL-6	Interleukin-6
MDA	Malondialdehyde
MPS	Microplastics
Nf-κb	Nuclear transcription factor-κB,
Nrf2	Nuclear Factor erythroid 2-Related Factor 2
PBS	Phosphate Buffered Saline
PCR	Polymerase Chain Reaction
PS-MPS	Polystyrene microplastic
PS-NP	Polystyrene nano microplastics
PVDF	Poly(vinylidene fluoride)
ROS	Reactive oxygen species
SDS-PAGE	Sodium Dodecyl Sulfate PolyAcrylamide Gel Electrophoresis
TEMED	Tetramethylethylenediamine
WB	Westernblot
